# Arabic version of the Australian type 2 diabetes risk assessment tool (AUSDRISK): translation and validation

**DOI:** 10.1186/s13104-022-06200-2

**Published:** 2022-09-22

**Authors:** Hassan Farag Mohamed Farag, Eman Anwar Sultan, Ehab Elrewany, Basem Farouk Abdel-Aziz

**Affiliations:** 1 Department of Tropical Health, High Institute of Public Health, Alexandria University, Alexandria, Egypt; 2 Department of Community Medicine, Faculty of Medicine, Alexandria University, Alexandria, Egypt; 3 Department of Health Administration and Behavioral Sciences, High Institute of Public Health, Alexandria University, Alexandria, Egypt

**Keywords:** T2DM, AUSDRISK Arabic Version, Forward–Backward translation, Screening, Reliability, Validity

## Abstract

**Objective:**

The current study aimed to translate the Australian Type 2 Diabetes Risk Assessment tool (AUSDRISK) into the Arabic language and evaluate the reliability and validity of the resultant Arabic version among Egyptians. The AUSDRISK was translated into Arabic language using the World Health Organization (WHO) forward and backward translation protocol. Using the WHO cluster sampling, a sample of 18+ years 719 Egyptians was randomly selected through a population-based household survey. Each participant was interviewed to fill the AUSDRISK Arabic version risk score and undergo confirmatory testing for fasting plasma glucose (FPG) and oral glucose tolerance test (OGTT). Test-retest reliability and convergent validity were computed.

**Results:**

Most of the study participants were physically active (60.5%) and females (69.3%). The Arabic version of the AUSDRISK reflected statistically significant perfect positive correlation (r = 1 and p < 0.01) for test re-test reliability as well as a significant moderate positive correlation with each of FPG (r = 0.48, p < 0.01) and OGTT (r = 0.52, p < 0.01) for the criterion-related (convergent) validity. The recalibrated noninvasive AUSDRISK Arabic version proved to be a simple, reliable, and valid predictive tool, and thereof, its employment for opportunistic mass public screening is strongly recommended. This can reduce diabetes mellitus Type 2disease burden and health expenditure.

## Introduction

The global prevalence of diabetes mellitus is rapidly increasing as a result of urbanization, population aging and associated lifestyle changes. In 2021, the International Diabetic Federation (IDF) has estimated that 537 million adults (20–79 years) worldwide had DM (10.5% of the population in this age group), rising to 643 million (11.3%) and 783 million (12.2%) by 2030 and 2045 respectively. About 75% of them were living in low- and middle-income countries (LMICs). Egypt ranks the 10th position of countries with the highest prevalence and is expected to become in the 9th position 2045, the number of diabetic Egyptians has been estimated to be 10.9 million and expected to reach 13 million and 20 million by 2030 and 2045 respectively [1].

T2DM accounts for 90% of all DM cases. Development and progress of T2DM is influenced by multiple variables dichotomized into modifiable (obesity, physical inactivity, consumption of unhealthy diet, high blood pressure and smoking) and non-modifiable (age, family history, ethnicity, and genetics) risk factors [2]. The purpose of screening for DM is to identify asymptomatic individuals who are likely to have DM for further prophylactic intervention. The IDF has indicated that lifestyle modification by physical activity and/or healthy diet can prevent or delay the onset of T2DM [3].

Screening for DM has relied for long time on invasive, inconvenient, and expensive techniques including blood sampling for measurement of fasting plasma glucose (FPG), the 2-hour oral glucose tolerance test (OGTT), or the glycated hemoglobin (HbA1c) [4]. Several non-invasive screening risk score charts have been developed, tested, and proved to be feasible, less time consuming, and cost effective in detecting T2DM in numerous countries [5].

Instead of blood sampling, the scientists have come up with risk assessment scoring; the rationale of which implies combining set of the most effective behavioral and biological risk factors to create a scaled instrument for dual screening and predictive functions. Several risk scores have been derived worldwide. Among these are the American Diabetes Association Diabetes Risk Test (ADADRT) [6], the Finnish Diabetes Risk Score (FINDRISK) [7], the Canadian Diabetes Risk Questionnaire (CANRISK) [8], the German Diabetes Risk Score (GDRS) [9], and the Australian type 2 Diabetes Risk assessment tool (AUSDRISK) [10].

Testing the validity and reliability of the proposed screening risk tool before their usage is mandatory. Validity indicates whether the tool actually measures whatever it is developed, designed, and intended to measure, while reliability refers to the consistency of the results provided by the tool whether it provides the same results every time of its use, in other term repeatability [11].

In 2008, composed of 10 risk factors (namely age, gender, country of birth, family history of diabetes, history of high blood glucose, hypertension, smoking status, fruit and vegetable intake, physical activity, and waist circumference), the AUSDRISK has been developed by the Australian Commonwealth Department of Health and Ageing in a bid to estimate the probability of T2DM development within the next 5 years [10]. In a cross-sectional study, AUSDRISK performance was evaluated and endorsed its high sensitivity (81.3%) and specificity (57.7%) in screening T2DM among participants aged 25–74 years and not previously known to be diabetic before [12].

The high prevalence and burden of T2DM among Egyptians has urged us to test the reliability and validity of a recalibrated Arabic version of the AUSDRISK in the early prediction of risk of T2DM development for early preventive intervention.

## Main text

### Materials and methods

The current study aimed to translate the AUSDRISK into Arabic language and test the reliability and validity of the Arabic version among Egyptians.

#### Phase 1: development of the Arabic version of the AUSDRISK

To develop the Arabic version of AUSDRISK the original tool was translated into Arabic according to the WHO forward-back translation protocol [13]. The initial step of the translation protocol (forward translation) was done by two independent bilingual native Arabic speakers. One of them was a health professional to be familiar with the terminology and the scope of the instrument. Two translations were checked by a tripartite bilingual expert panel that checked for any significant discrepancies. Differences were decided to be merely expressional/stylistic. The two translations were judged to be semantically equivalent. Inconsequential differences were resolved by the committee by electing the simpler wording/phrase to produce an Arabic version for backward translation. The Arabic translated version was back translated into English by two independent bilingual native English speakers who had no knowledge about the questionnaire. The back translation was examined by the bilingual expert panel to resolve any discrepancies between the two back translations and the original English version. No genuine discrepancies as the noted differences were only idiomatic. The final Arabic version of the AUSDRISK was settled upon by the committee.

The AUSDRISK Arabic version consisted of 9 variables instead of 10 of its original one. The question related to ethnicity and country of birth was removed; Egyptians have no different ethnicities. The total score of the added-up scores of the 9 variables ranged between 0 and 34 points and was scaled as: mild risk (≤ 4 points), moderate risk (5–10 points) and severe risk (≥ 11 points).

#### Phase 2

To test the created AUSDRISK Arabic version, a cross-sectional population-based household study was conducted using the WHO cluster sampling technique in Damanhur district, El Behera Governorate, Egypt. 719 Adults aging 18 years or more and not known to be diabetics were recruited to participate in the study with exclusion of pregnant females or those who had advanced decompensated organ disease. Each participant was interviewed face to face to fill the Arabic version of the AUSDRISK (twice, 2 weeks apart) and invited to perform fasting plasma glucose (FPG), plasma glucose level after no caloric intake for at least 8 h and oral glucose tolerance test (OGTT), plasma glucose level 2 h after intake of 75 g anhydrous glucose dissolved in water used as a glucose load. Method used to determine fasting and post prandial plasma glucose levels is Roche Cobas C3112017—Standard.

Sampling approach was adopted as the following:

Stage 1: Using simple random sampling, Damanhur district was selected among the 15 districts of El Behera governorate.

Stage 2: In Damanhur district, WHO cluster sampling technique was adopted as follows:

- A sum of 30 clusters were identified to be involved in the study.
- Proportional allocation was used in selecting the clusters whereby two thirds (20 clusters) and one third (10 clusters) were rural and urban clusters respectively.
- From each cluster ten households were selected and involved in the study.
- Abandoned and closed households were ignored.
- Within each household, all eligible adults were included in the study.

#### Statistical analysis

The collected data were coded, revised, cleaned, tabulated, and analyzed through IBM SPSS Statistics version 26 using appropriate statistics [14]. The descriptive statistics were calculated for qualitative and quantitative data to describe the study population. The analytic statistical tests comprised; the AUSDRISK Arabic version test re-test reliability and criterion related (convergent) validity calculated using Pearson correlation coefficient (r).

## Results

Table 1 distributed the studied participants by the Arabic version of the 9-item AUSDRISK score. The calculated total score ranged from 0 to 31 with a mean total score of 9.57 ± 6.06, categorized 21.5%, 39.4%, and 39.1% of the participants at mild (≤ 4), moderate [5–10], and severe (≥ 11) risk. Most of the participants were non-smoker (88.3%), females (69.3%), had no history of high blood glucose level (92.8%), not used to take hypotensive medications (87.6%) and used to eat fruits and vegetables daily (70.0%). The participants inclined moderately towards weekly 2.5 h of physical activity (60.5%) and family history of DM among their first-degree relatives (40.3%) and mildly towards age group < 35 years (45.9%) and normal waist circumference of < 102 cm for male and < 88 cm for female (41.7%).

**Table 1:** Participants’ AUSDRISK Arabic version scores

			Total (n = 719)	
			N	%
*1- Your age group (years)*				
< 35		0 point	330	45.9
35-		2 points	139	19.3
45-		4 points	103	14.3
55-		6 points	97	13.5
≥ 65		8 points	50	7.0
*2- Your gender*				
Female		0 point	498	69.3
Male		3 points	221	30.7
*3- Have either of your parents, or any of your brothers or sisters been diagnosed with diabetes (type 1 or type 2)?*				
No		0 point	429	59.7
Yes		3 points	290	40.3
*4- Have you ever been found to have high blood glucose (sugar)?*				
No		0 point	667	92.8
Yes		6 points	52	7.2
*5- Are you currently taking medication for high blood pressure?*				
No		0 point	630	87.6
Yes		2 points	89	12.4
*6- Do you currently smoke cigarettes or any other tobacco products on a daily basis?*				
No		0 point	635	88.3
Yes		2 points	84	11.7
*7- How often do you eat vegetables or fruits?*				
Every day		0 point	503	70.0
Not every day		1 point	216	30.0
*8- On average, would you say you do at least 2.5 h of physical activity per week?*				
Yes		0 point	435	60.5
No		2 points	284	39.5
*9- Waist measurement (in cm) (below the ribs at the level of the navel while standing)*				
Male < 102 cm / female < 88 cm		0 point	300	41.7
Male 102–110 cm / female 88–100 cm		4 points	233	32.4
Male > 110 cm / female > 100		7 points	186	25.9
*Total score*		Low risk ≤ 4	155	21.5
Min – Max	0–31	Moderate risk 5–10	283	39.4
Mean ± SD	9.57 ± 6.06	Severe risk ≥ 11	281	39.1

Test re-test reliability of the recalibrated tool was reflected by the statistically significant perfect positive correlation (r = 1 and p < 0.01) between scores of all participants interviewed two weeks apart. Criterion-related (convergent) validity was reflected by significant moderate positive correlations between the Arabic AUSDRISK score and FPG (r = 0.48 and p < 0.01) and OGTT (r = 0.52 and p < 0.01) as well.

## Discussion

The current study worth arises from commonness of DM in Egypt which recorded 10.9 million DM patients in 2021 and is expected to be 13 and 20 million by 2030 and 2045 respectively. This ranked Egypt the tenth in the list of top 10 global countries with highest DM prevalence in 2021 and is expected to advance it to the ninth position by 2045 [1]. Screening is the backbone of T2DM preventive strategy. It aims to screen the asymptomatic apparently healthy people to find out undiagnosed T2DM, pre-diabetes (PDM) and those who are vulnerable (at risk) to get T2DM. This should be followed by appropriate non-pharmaceutical and/ or pharmaceutical intervention to prevent or delay disease occurrence [15].

Screening for DM became dependent on the development of screening risk score charts and test their validity and reliability to replace the invasive blood sampling for measurement of FPG, OGTT or HbA1c [4, 5]. Several risk scores have been derived worldwide either as a new original tool like ADADRT [6], FINDRISK [7], CANRISK [8], GDRS [9], and AUSDRISK [10] or by translation and adaptation of the original tool in different population. The FINDRISK and CANRISK were translated and calibrated to be used among Brazilians [16, 17]. The ADADRT was translated and validated among the Malaysians [18]. The CANRISK was translated into different languages and tested for its validity and reliability, e.g., among the Arab [19] and the Chinese population [20].

Candidacy of the AUSDRISK for the current study has been explained on the ground of the similarity between the multiethnic Australian people from which the AUSDRISK has been derived and the mixed nature of the Egyptian society. Also, the previous utilization of the original tool in screening surveys in Egypt empowered and encouraged the recalibration process to develop an Arabic version for the Egyptian population [21, 22].

In the present study, as per the AUSDRISK scores, 39.1%, 39.4%, and 21.5% of the participants were at high, moderate, and low T2DM risk, respectively. These findings are not identical to most of the previous studies conducted outside [10, 12, 23] and inside [21]. Egypt in which the noted proportion of the participants at high T2DM risk was at least 50%. Involvement of younger age group and females stood behind this discrepancy; 45.9% and 69.3% of the participants were below the age of 35 years and females respectively. The older the age the more the AUSDRISK score and higher the risk of T2DM and vice versa. Previous studies including younger age segments, young age was linked to a lower proportion of participants classified as high T2DM risk. For instance, a study among Egyptian and Malaysian students at Tanta University in Egypt demonstrated severe risk of diabetes was found among 17.7% and 9.9% of Egyptian and Malaysian students respectively. The age of Egyptian and Malaysian students ranged from 20 to 24 years [22]. Another example is an Australian study included participants ≥ 18 years and demonstrated that 30.8% of participants were categorized as high T2DM risk. and the results indicated that the predicted 5-year risk significantly increases with age. Percentages of those at high risk were 0.29%; 1%; 4.96; 7.86 and 16.73% for age groups (18–34); (35–44); (45–54); (55–64); and (≥ 65) respectively [24]. These findings bring to light the simplicity and practicality of the AUSDRISK screening power.

Present study revealed statistically significant perfect positive correlation for the test re-test reliability of the tool. Convergent (criterion-related) validity was demonstrated by the positive significant moderate correlation between the tool score and FPG and OGTT. Reliability and convergent validities were higher than those recorded in other cross-sectional studies in Jordan and Saudi Arabia, as the ARABRISK score reflected high agreement for test-retest reliability (ICC3,1 = 0.98, CI = 0.97-0.99) and correlated significantly with FPG (r = 0.3, P = 0.01). The ARABRISK was developed and reflected high reliability and validity in Jordan and Riyadh [19]. The utility of AUSDRISK to identification and follow up of T2DM risk groups after lifestyle modification was proved by previous research [23]. This encouraged the application of the Arabic AUSDRISK version among Egyptians in screening and preventive programs.

## Conclusions and recommendations

The Arabic AUSDRISK was proved to be valid and reliable tool to be used among Egyptians as a screening tool. It is recommended to apply the AUSDRISK Arabic version in based opportunistic screening at Family Medicine and Primary Health Care centers to identify population at risk of T2DM. Also, the inclusion of AUSDRISK Arabic version as a screening tool during the mass public survey and health services.

### Limitations

This study is restricted to Behera Governorate in Egypt. To be generalizable to Egypt the study needs to be replicated to represent the 27 Egyptian Governorates. Generalizability to the Arab World needs extending the study to represent the 22 member countries of the Arab league. Generalizability to Arab speaking communities all over the globe needs more extensive research efforts.

## Data Availability

Data are available from the third co-author on reasonable request.

## Abbreviations

AUSDRISK: Australian Type 2 Diabetes Risk Assessment Tool
DM: Diabetes Mellitus
FPG: Fasting Plasma Glucose
HbA1c: Glycated Hemoglobin
IDF: International Diabetic Federation
LMIC: Low- and Middle-Income Countries
OGTT: Oral Glucose Tolerance Test
PDM: Pre-diabetes
r: Pearson Correlation Coefficient
SD: Standard Deviation
T2DM: Type 2 Diabetes Mellitus
WHO: World Health Organization
